# Therapy Dogs for Anxiety in Children in the Emergency Department: A Randomized Clinical Trial

**DOI:** 10.1001/jamanetworkopen.2025.0636

**Published:** 2025-03-14

**Authors:** Heather P. Kelker, Huma K. Siddiqui, Alan M. Beck, Jeffrey A. Kline

**Affiliations:** 1 Department of Emergency Medicine, Indiana University School of Medicine, Indianapolis; 2 Center for Human Animal Bond, Purdue University School of Veterinary Medicine, Lafayette, Indiana; 3 Department of Emergency Medicine, Wayne State University School of Medicine, Detroit, Michigan

## Abstract

**Importance:**

Prior evidence suggests that the use of therapy dogs in emergency care reduces anxiety in adults, but no trial has tested the use of therapy dogs in emergency care of children.

**Objective:**

To examine whether adjunctive use of therapy dogs in standard child-life therapy reduces child-reported and parent-reported child anxiety in a pediatric emergency department (ED).

**Design, Setting, and Participants:**

This randomized clinical trial was conducted from February 1, 2023, to June 30, 2024, at an academic pediatric ED. Children (aged 5-17 years) with suspected moderate to high anxiety were included.

**Intervention:**

All participants received standard child-life therapy, and the intervention group was randomly assigned to have exposure to a therapy dog and handler for approximately 10 minutes.

**Main Outcomes and Measures:**

Anxiety was measured using the 0- to 10-point FACES scale (with 0 indicating no anxiety and 10 indicating very severe anxiety) and salivary cortisol concentrations. Measurements were obtained at baseline (T0), 45 minutes (T1), and 120 minutes (T2) for both child and parents.

**Results:**

A total of 80 patients (mean [SD] age, 10.9 [3.8] years; 45 [56%] female) were enrolled (40 in the control group and 40 in the intervention group). At T0, the mean (SD) FACES scores were 5.4 (2.8) for child report and 6.4 (2.4) for parent report; the means were not different between groups. From T0 to T1, child-reported anxiety changed by a mean (SD) of −1.5 (3.4) points in the control group vs −2.7 (2.5) points in the intervention group (*P* = .02, Mann-Whitney *U* test); similarly, mean (SD) parent-estimated child anxiety changed by −1.8 (2.7) points in the control group vs −3.2 (2.3) points in the intervention group (*P* = .008). A total of 9 children (23%) in the control group had a greater than 2.5-point decrease in FACES score vs 18 (46%) in intervention group (*P* = .04, Fisher test). At T2, mean (SD) child-reported FACES scores decreased to 3.6 (3.4) points in the control group and 3.0 (2.7) points in the intervention group (*P* = .70). A total of 14 control participants (35%) received ketamine, midazolam, lorazepam, or droperidol vs 7 (18%) in the intervention group (*P* = .08, Fisher test). Child and parent salivary cortisol decreased from T0 to T1 in both groups but was not different between groups. Parental salivary cortisol was significantly consistently higher than their children’s salivary cortisol (*P* < .001, unpaired *t* test, for comparisons of child vs parent at T0 and T1 in both groups).

**Conclusions and Relevance:**

This study of adjunctive use of therapy dogs in standard child-life therapy found a modest but significantly greater reduction in both child-reported and parental-reported child anxiety in the pediatric ED for the intervention vs control group. These findings support the use of therapy dogs to help reduce pain and anxiety without the use of chemical or physical constraint.

**Trial Registration:**

ClinicalTrials.gov Identifier: NCT03784573

## Key Points

**Question:**

Can adjunctive use of therapy dogs in child-life therapy reduce both child-reported anxiety and parental report of child anxiety in the pediatric emergency department (ED)?

**Findings:**

In this randomized clinical trial of 80 children, the use of therapy dogs afforded greater reduction in both child-reported and parent-reported child anxiety in the pediatric ED. Salivary cortisol levels decreased in children and parents, but parental salivary cortisol levels were consistently significantly higher than those in children.

**Meaning:**

These findings suggest that therapy dogs can reduce child-reported anxiety and parental perception of child anxiety in the pediatric emergency department.

## Teaser

This randomized clinical trial assesses whether interaction with a therapy dog reduces anxiety among children in a pediatric emergency department.

## Introduction

Children who visit the emergency department (ED) often experience psychological stress and anxiety. This anxiety may be amplified by usual care processes that cause pain or fear, such as phlebotomy or intravenous access. Approximately 15% of children require chemical or physical interventions to allow care processes to continue.^1,2^

Many children who visit the ED have chronic medical and psychiatric conditions. Anxiety is both more common and more severe in children with chronic disease, psychiatric diagnoses, spectrum disorders, and cognitive impairment.^3,4,5,6^ Anxiety worsens short- and long-term outcomes of many chronic conditions that require frequent ED visits, such as asthma and sickle cell disease.^3,4,6^ Additionally, parental perception of their child’s fear can amplify the stress response, negatively affecting the child’s pain perception and recollection of the entire ED visit, which in turn can contribute to long-term threat perception and future avoidance of emergency care.^7,8,9,10,11,12^

To improve the child and parental experience, pediatric EDs employ certified child-life specialists to help mitigate the fear and anxiety a child experiences by using play therapy, visual and auditory distractions, and other situationally appropriate techniques at a developmentally appropriate level. The Child Life Council of the American Academy of Pediatrics has designated child-life programs as a “standard in most large pediatric centers and even on some smaller pediatric inpatient units to address the psychosocial concerns that accompany hospitalization and other health care experiences.”^13^

Prior literature^14,15,16^ demonstrates that human psychological stress can be reduced with exposure to animals. Studies^16,17^ have found reduction in stress using therapy dogs in more than 1 health care setting. In nonemergency care settings, therapy dogs have demonstrated a larger effect size on anxiety reduction for children who are most vulnerable to anxiety and stress, such as those with psychiatric disorders, spectrum disorders, or chronic medical conditions.^14,18,19,20^ However, in a systematic review and meta-analysis, Gaudet et al^21^ pooled data from 5 randomized clinical trials in adults in the emergency care setting and found no consistent benefit in anxiety or distress with pet therapy.

To our knowledge, no study has investigated the effect of therapy dog exposure in the emergency care of children. The main study hypothesis of this work is that pediatric patients with moderate to high anxiety will have a significant reduction in patient-reported anxiety, using the visual FACES scale, after interacting with a therapy dog compared with usual care. The secondary hypothesis stated that single exposure to a therapy dog and handler reduces caregiver perception of the child’s anxiety and pain.

## Methods

### Overview

This registered, single-center, hypothesis-testing randomized clinical trial evaluated the effectiveness of usual care with a child-life specialist plus approximately 10 minutes with a therapy dog and handler, compared with exposure to a child-life specialist alone, on patient-reported outcome of anxiety. Changes to the trial from the registered protocol included not collecting data on the child’s self-reported pain and changing age to include 5 to 17 years due to concern for cooperation with 4-year-olds. This trial was conducted from February 1, 2023, to June 30, 2024, at Riley Children’s Hospital in Indianapolis, Indiana, and was approved by the Indiana University School of Medicine Institutional Review Board. Participants were recruited by written informed consent from parents and assent from children by qualified study personnel who explained the protocol in a single-occupancy ED room. The trial followed the Consolidated Standards of Reporting Trials (CONSORT) reporting guideline. The institutional review board–approved trial protocol is available in Supplement 1.

We assumed that the current best practice for anxiety reduction is the intervention of child-life specialists. Riley Children’s Hospital requires that child-life specialists undergo rigorous training and maintenance of certification. All human participants were unpaid volunteers, and the study was funded by the Human Animal Bond Research Institute in conjunction with Wayne State University. All dogs and handlers were therapy certified. Handler and dog training and welfare were conducted in strict accordance with the published guidelines of the Pet Partners Therapy Animal Program.^22^ Because the dogs were not research subjects, the Institutional Animal Care and Use Committee deemed this study as outside the scope of their authority for approval. Other than the shutdown of the therapy dog program during the COVID-19 pandemic (2020-2022), there were no specific challenges to using therapy dogs in the ED because the Riley Children’s Hospital already has a therapy dog program in the hospital.

### Patient Participants and Trial Design

**Figure 1.**
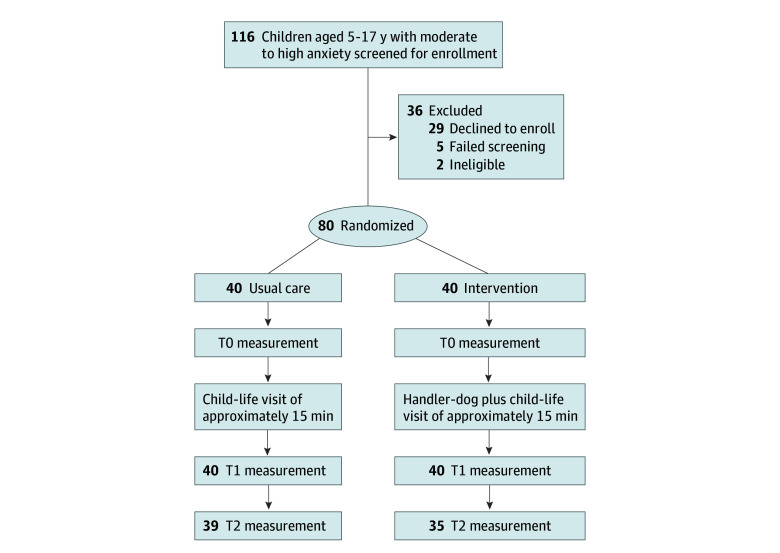
CONSORT Flow Diagram T0 indicates baseline; T1, 45 minutes; T2, 120 minutes.

The clinician (nurse or physician) with primary patient care responsibilities used assessment of anxiety as a method to screen for potential participants, with participants with moderate to high anxiety (score of ≥6 on the 0- to 10-point FACES scale, with 0 indicating no pain and 10 indicating very severe pain) aged 5 to 17 years approached for assent and informed consent. Exclusion criteria included any medical emergency requiring immediate intervention, violent behavior, intoxication (parent or child), severe physical or cognitive limitations that would preclude any sensory interaction with dogs, fear of or prior adverse reaction to dogs, dog allergy, or chief concern of asthma exacerbation with dogs as a known trigger. Demographic data, including reported race and ethnicity, were obtained from the electronic health record by qualified study personnel. Race and ethnicity are important to document to determine whether children have responses to the stimulus that vary based on their cultural background. The protocol, following the CONSORT diagram model, is shown in Figure 1.

The randomization schedule was produced in advance using a computerized generator with a seed of 74243484, with an equal number of controls (child-life therapy only) and intervention patients (child-life handler and therapy dog) opportunities in each group. The random sequence was only seen by the research coordinator (H.K.S.) after informed consent. When control patients were enrolled, no dog was in the department. The enrollment of patients in the control and intervention groups took place during the same time of day (10 am to 4 pm), and patients were not grouped into categories of similar diagnosis for comparison.

All children received exposure to standard child-life therapy (eg, age-appropriate explanations, distraction, and comfort) and were randomized to receive either nothing additional (control) or added exposure to a therapy dog and handler for approximately 10 minutes (intervention). Research procedures did not interfere with examinations or medical processes, such as venipuncture or imaging. In the event of an interruption, the dog and handler left the room and returned later to complete the session. Per protocol measurements included child- and parent-observed scales and saliva sampling, obtained at baseline (T0, before meeting the dog and handler and, as much as practicable, before meeting the child-life specialist or before any painful procedure or care); then again at 45 minutes after exposure to the dog and handler and/or child-life specialist (T1); and at approximately 120 minutes or as close as possible to discharge (T2). At T0, T1, and T2, one consenting caregiver (chosen by the guardian) completed the FACES scale for their perceptions of their child’s anxiety, and they also marked their overall perception of stress of the child on a 1- to 100-mm visual analog scale (with 1 indicating no stress and 100 indicating highest possible stress) and their perception of their child’s severity of pain on a 0- to 10-point pain scale (with 0 indicating no pain and 10 indicating worst pain of life). To reduce the child’s cognitive workload, we asked the child to rate his or her anxiety on the FACES scale only at T0, T1, and T2, given our belief that asking a child to rate both pain and anxiety on a FACES scale may have been too confusing for a young child. The FACES scale has been validated previously as a method of assessing anxiety and is the most plausible scale to use in children aged 5 to 17 years.^23,24,25^ A qualified study associate (H.K.S.) obtained the patient’s heart rate from a finger probe pulse oximeter from the child (or from the electronic health record) at T0, T1, and T2 and salivary cortisol from the child and parent or guardian using the passive drool technique, which was analyzed using a vendor with independent analysis (Salimetrics) at T1. The 45-minute interval (T0 to T1) has been found to be the optimal time for salivary cortisol to change in response to therapy dog exposure and is also justified by prior work in adults.^26,27^ Children randomized to the control group had the opportunity to see a handler and dog after the T2 measurement. Study personnel kept notes on the quality of the child and dog handler interaction, and handlers reported their impression of the degree of engagement of the patient with the dog as follows: slightly (2 or 3 touches), moderate (>3 touches), engaged (touching constantly or touching for >3 minutes), and highly engaged (talking to animal, playing, or touching the entire time).

Medication use for anxiolysis was also documented, with midazolam being the most commonly used medication in this pediatric ED. Ketamine is used for facilitation of painful procedures at sedative doses, especially in some highly anxious young children, who would not tolerate a procedure with standard anxiolytic plus local anesthetic. Any benzodiazepine, haloperidol, and droperidol use was also documented for anxiolysis. We did not record use of clonidine.

### Sample Size

The primary outcome was the change in child-reported and parent-reported child anxiety on the FACES scale from T0 to T1. Extrapolating from our work in adults, we set the clinically significant reduction in anxiety as requiring a greater than 2-point (20%) decrease in anxiety at T1 compared with usual care (a child-life specialist), expecting an SD of 3. With α = .05 and β = .20, this required 37 pairs; 20% is justified from work in adults.^28^ Accordingly, the sample was set at 40 per group (N = 80) with complete data.

### Statistical Analysis

Data were analyzed with an intention-to-treat design.^29^ FACES data were tested for normality with a Shapiro-Wilk test and did not uniformly pass as normally distributed. Therefore, the primary aim, change in child-reported and parent-reported child FACES scores between T0 and T1, was compared between groups using the Wilcoxon rank sum test. Prior work in adults has suggested that a 2.5-point change on the FACES scale is clinically important; therefore, we also assessed the proportion of children who had a greater than 2.5-point change with a Fisher exact test.^28^ We considered the use of ketamine, haloperidol, droperidol, or midazolam for anxiolysis and compared the frequency of their administration with a Fisher exact test. To further examine group changes from T0 through T2, we also performed a 2-way, repeated-measures, mixed-effects analysis of variance (ANOVA) to compare group effect and time × group interaction on the change in perceived FACES scales as previously described.^1^ We compared anxiety scores from clinicians, parents, and children with a 1-way ANOVA followed by a Tukey post hoc test and measured strength of correlation among clinician, parent, and child perceptions on the FACES scale with a Spearman rank coefficient. Salivary cortisol concentrations were compared using a paired *t* test from T0 to T1 and an unpaired *t* test applied to the T0 to T1 change in salivary cortisol concentrations between groups. The primary measurement to define a significant reduction includes significance at α = .05 for comparison of the median FACES score at the first time point. Quantitative data analyses and graphing were performed using GraphPad Prism for Windows, version 10.0.0 (GraphPad Software).

## Results

### Enrollment and Engagement

A total of 80 patients (mean [SD] age, 10.9 [3.8] years; 45 [56%] female and 35 [44%] male; 2 Asian [3%], 24 Black [30%], 53 [66%] White, and 1 [1%] reporting >1 race) were enrolled (40 in the control group and 40 in the intervention group) (Figure 1). In the control group, 39 had complete data at T2, with 1 missing because of unexpected early discharge; in the intervention group, 35 had complete data at T2. In the dog therapy group, the mean (SD) time from T0 to T1 was 63 (17) minutes, and the mean (SD) time from T0 to T2 was 150 (52) minutes. In the control group, the mean (SD) time from T0 to T1 was 45 (13) minutes, and the mean (SD) time from T0 to T2 was 133 (76) minutes. Table 1 presents clinical and demographic information by randomized group. The largest difference in categorical features at enrollment was the 18% (95% CI, −3% to 37%) difference in use of prescribed medications at home. Thirty-six children (45%) had 1 or more chronic medical condition, 12 (15%) had a recorded psychiatric diagnosis, and 20 (25%) reported they had visited the ED within the previous 6 months. All children assigned to the handler and dog intervention interacted with the dog to some degree. Field notes generally reflected positive interactions from the child and family participants.

**Table 1.** Clinical and Demographic Characteristics of the Enrolled Children

Characteristic	No. (%) of children^a^	
	Dog therapy (n = 40)	No dog therapy (n = 40)
Age, mean (SD), y	11.7 (4.0)	10.1 (3.5)
Data to T1	40 (100)	40 (100)
Data to T2	39 (98)	35 (88)
Sex		
Female	25 (63)	20 (50)
Male	15 (38)	20 (50)
Race^b^		
Asian	1 (3)	1 (3)
Black	12 (30)	12 (30)
White	26 (65)	27 (68)
>1 Race	1 (3)	0
Hispanic or Latino ethnicity	5 (13)	4 (10)
No significant medical history	16 (40)	20 (50)
Prior psychiatric diagnosis	5 (13)	7 (18)
Prescribed medication at home	31 (78)	24 (60)

Abbreviations: T0, baseline; T1, 45 minutes.

^a^ Unless otherwise indicated. Percentages may not sum to 100 due to rounding.

^b^ By patient report.

### Comparison of Anxiety Assessments

Child anxiety at T0 was assessed by nurses in 20 children (25%) and by physicians in the remainder. Anxiety levels on the 0- to 10-point FACES scale for clinicians, parents, and children indicate that clinicians and parents both overestimated anxiety compared with child self-assessment (mean [SD] anxiety scores, 7.2 [1.3] for clinicians, 6.4 [2.2] for parents, and 5.5 [2.8] for children; *P* = .08 for clinicians vs parents, *P* = .01 for parent vs child, and *P* < .001 for child vs clinician, Tukey post hoc test), with the overall strength of agreement being weak for clinicians (Spearman ρ = 0.17; 95% CI, 0-0.73) vs parents (Spearman ρ = 0.36; 95% CI, 0.16-0.54) or children (Spearman ρ = 0.10; 95% CI, 0-0.30); however, parents had a higher strength of agreement with their children (Spearman ρ = 0.37; 95% CI, 0.16-0.53).

### Effect of Handlers and Dogs on the Primary Outcome

**Figure 2.**
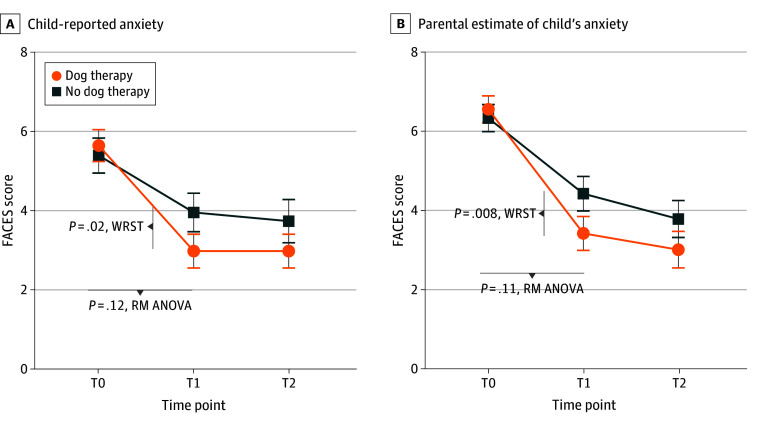
Child- and Parent-Reported Anxiety Reported on the FACES Scale A score of 0 indicates no pain and 10 indicates very severe pain. Error bars indicate SDs. RM ANOVA indicates repeated-measures analysis of variance (see Statistical Analysis section for further explanation); WRST, Wilcoxon rank sum test.

Figure 2 shows the main findings, and Table 2 presents additional detail. At T0, mean FACES scores did not differ between groups for either the child reports (mean [SD] score, 5.4 [2.8]) or parent reports (mean [SD] score, 6.4 [2.4]). Compared with T0, at T1, child anxiety decreased in both groups, with a mean (SD) change of −1.5 (3.4) points in the control group vs −2.7 (2.5) points in the intervention group (*P* = .02, Mann-Whitney *U* test). Similarly, parental report of child anxiety changed by a mean (SD) of −1.8 (2.7) points in the control group vs −3.2 (2.3) points in the intervention arm (*P* = .008). However, when the FACES scale data were compared with a repeated-measures ANOVA, the group × time interaction *P* values were not significant for either the child participants (*P* = .11) or the parent participants (*P* = .12). A total of 9 children (23%) in the control group had a greater than 2.5-point decrease in FACES score compared with 18 (46%) in the intervention arm (*P* = .04, Fisher exact text). By T2, mean (SD) FACES scores from children decreased to 3.6 (3.4) in the control group vs 3.0 (2.7) after intervention (*P* = .70).

**Table 2.** Measurements of Parameters Associated With Stress in Child and Parent Participants

Measurement and time	Mean (SD)	
	Dog therapy	No dog therapy
**Child**		
Anxiety score, points^a^		
T0	5.4 (2.8)	5.5 (2.8)
T1^b^	3.0 (3.0)	4.0 (3.1)
T2	3.3 (2.9)	3.5 (3.4)
Heart rate, beats/min		
T0	95 (25)	92 (19)
T1	90 (27)	90 (19)
T2	90 (28)	93 (22)
Systolic blood pressure, mm Hg		
T0	118 (17)	116 (14)
T1	110 (14)	111(13)
T2	110 (19)	110 (14)
**Parent**		
Anxiety score, points^a^		
T0	6.6 (2.1)	6.3 (2.2)
T1^c^	3.4 (2.7)	4.1 (2.8)
T2	3 (2.5)	3.8 (2.9)
Stress score, points^d^		
T0	65 (27)	62 (22)
T1	34 (24)	35 (26)
T2	34 (24)	33 (29)
Pain rating of the child, points^e^		
T0	5 (3.4)	6.1 (2.9)
T1	2.9 (2.9)	3.8 (3.1)
T2	2.1 (2.9)	3.4 (2.9)

Abbreviations: T0, baseline; T1, 45 minutes; T2, 120 minutes; VAS, visual analog scale.

^a^ Anxiety was measured using the 0- to 10-point FACES scale (with 0 indicating no pain and 10 indicating very severe pain).

^b^ *P* = .02 for comparison of dog therapy vs no dog therapy (Wilcoxon rank sum test).

^c^ *P* = .008 for comparison of dog therapy vs no dog therapy (Wilcoxon rank sum test).

^d^ Overall perception of stress of the child was measured on a 1- to 100-mm VAS (with 1 indicating no stress and 100 indicating highest possible stress).

^e^ Parental perception of child’s severity of pain was measured on a 0- to 10-point pain scale (with 0 indicating no pain and 10 indicating worst pain of life).

### Medication Administration

After child-life exposure, 14 control participants (35%) were administered at least 1 dose of ketamine, midazolam, lorazepam, or droperidol vs 7 (18%) in the intervention group (*P* = .08, 2-sided Fisher exact test). No patient received haloperidol.

### Other Measurements

Table 2 shows no baseline differences between groups in vital signs, parental assessment of overall stress, or parental assessment of their child’s pain. There were no significant changes in vital signs, and although parent assessment of child anxiety and pain decreased, this was similar in both groups. No adverse events or harms occurred during the study.

### Salivary Cortisol

The eTable in Supplement 2 presents the mean values of salivary cortisol, and we found no significant differences between groups for the means at T1 and T2. Children with exposure to the dog had a significant mean (SD) decrease in salivary cortisol from T0 to T1 (0.04 [0.19] μg/dL; *P* = .02, paired *t* test), whereas children in the control group had no change from T0 to T1 (0.03 [0.10] μg/dL; *P* = .58, paired *t* test). However, no mean (SD) difference was found when the change in salivary cortisol concentration from T0 to T1 was compared between the child participants who received dog therapy and those who did not receive dog therapy (0.03 [0.10] μg/dL; *P* = .74, unpaired *t* test). For parents, salivary cortisol decreased similarly in both groups without any difference in the mean (SD) change from T0 to T1 change (0.21 [0.47] μg/dL; *P* = .71). A salient post hoc finding was that parental salivary cortisol was consistently higher than their children (*P* < .001, unpaired *t* test, for comparisons of child vs parent at T0 and T1 in both groups).

## Discussion

This clinical trial demonstrates novel evidence that animal-assisted therapy, adjunctive to child-life therapy, can reduce both child patient and parental perception of anxiety in their child in the ED. We found that compared with usual care with child-life specialists, when anxious children were exposed to an additional experience with a certified therapy dog and handler, they reported a statistically significant decrease in anxiety 45 minutes later (T1). Simultaneously, the parental or guardian participants also reported significantly lower child anxiety with exposure to the therapy dog and handler. We found a reduced use of medications commonly used for behavioral control or to treat severe anxiety (midazolam, ketamine, lorazepam, and droperidol), although this change was not significantly different from the control group. We found no difference in salivary cortisol between groups, but we did observe that cortisol levels tended to be highest at T0 for both children and parents, suggesting a physiologic rationale to support the introduction of an animal-based intervention for anxiety reduction at the soonest convenience possible. The data also showed that parental salivary cortisol levels were consistently higher than in their children, which may reflect higher activation of the hypothalamic-pituitary-adrenal axis from anxiety in parents or an age-related artifact. We are unaware of any literature reporting age-based differences in salivary cortisol.

We believe this is the first randomized clinical trial of animal therapy in the pediatric ED to measure patient- and parent-reported outcomes for children. Prior studies have shown a positive effect of therapy dogs on pain, anxiety, and perceptions of experience interfacing with medical care in various settings.^14,15,16,17,30,31,32,33^ Several studies have reported data on therapy dogs in the ED setting; however, this is the first, to our knowledge, to focus on the pediatric population and the unique interplay of parent or guardian perceptions of pain and anxiety in children.^28,32,33^ The experience of children in the ED can produce strong feelings of fear and anxiety due to unfamiliarity, potentially noxious interventions necessary for medical care, and the inability to explain the circumstances to some young children at a developmentally appropriate level. Approximately 78% of children in the ED experience both pain and anxiety.^10,12,34^ One study conducted during 5 years in a tertiary urban ED found that 4.6% of all children and adolescents were physically or chemically restrained during their ED stay.^1^ Children with psychiatric diagnoses are at higher risk of receiving chemical sedation; one study found that 1 in 15 children in the ED with psychiatric conditions receive chemical sedation.^2^ Twelve children (15%) in our sample had an existing psychiatric condition, and these participants demonstrated similar improvements in their reported anxiety compared with children without diagnosed psychiatric conditions.

Nonpharmacologic methods to reduce pediatric pain and anxiety have included reliance on parental presence, art or music therapy, and distraction with varying degrees of effectiveness.^12,35,36,37,38^ The introduction of a familiar animal, such as a dog, previously certified by a rigorous process as friendly, tolerant, and warm can communicate a natural sense of safety, coherence, and comfort to a child that may exceed what can be expressed by emergency care clinicians, whom children often perceive as strangers. Child-life specialists are highly trained to help children and parents understand emergency care at a developmentally appropriate level. Our data provide initial evidence that the handler and therapy dog adjunct can further augment the goal of minimizing pain and anxiety without the use of chemical or physical restraint. The data from this work provide further support of the biophilia hypothesis in medicine.^19,20,39,40^

### Limitations

This study has some limitations. Blinding to the presence of a dog was not possible; therefore, this could be a source of bias and overestimation of effects. This study is generalizable only to EDs with capacity for therapy dog use and child-life specialists, and there is variability in the provision of standard child-life therapy, which could lead to potential bias or effect modification. Our data do not allow a specific inference about the magnitude of effect of therapy dogs in EDs that do not have child-life specialists present.

## Conclusions

In this randomized clinical trial, adjunctive use of therapy dogs to standard child-life therapy afforded modest but significantly greater reduction in both child-reported and parental-reported child anxiety in the pediatric ED for the intervention group compared with the control group. These findings provide initial evidence for the use of therapy dogs to minimize pain and anxiety without the use of chemical or physical restraint among pediatric ED patients.

## Notes

Supplement 1.
Trial Protocol

Supplement 2. **eTable.** Salivary Cortisol Measurements (μg/dL)

Supplement 3.
Data Sharing Statement

## References

[1] Vishnoi G, Feuer V. Looking back 5 years: restraint and intramuscular medication use for psychiatric patients in the pediatric emergency department of a tertiary care children’s hospital in New York. J Am Acad Child Adolesc Psychiatry. 2019;58(10):S181. doi:10.1016/j.jaac.2019.08.125

[2] Gerson R, Malas N, Mroczkowski MM. Crisis in the emergency department: the evaluation and management of acute agitation in children and adolescents. Child Adolesc Psychiatr Clin N Am. 2018;27(3):367-386. doi:10.1016/j.chc.2018.02.002 29933788

[3] Cobham VE, Hickling A, Kimball H, Thomas HJ, Scott JG, Middeldorp CM. Systematic review: anxiety in children and adolescents with chronic medical conditions. J Am Acad Child Adolesc Psychiatry. 2020;59(5):595-618. doi:10.1016/j.jaac.2019.10.010 31676391

[4] Downey VA, Zun LS. Identifying undiagnosed pediatric mental illness in the emergency department. Pediatr Emerg Care. 2018;34(2):e21-e23. doi:10.1097/PEC.0000000000001151 28441242

[5] Lytle S, Hunt A, Moratschek S, Hall-Mennes M, Sajatovic M. Youth with autism spectrum disorder in the emergency department. J Clin Psychiatry. 2018;79(3):22582. doi:10.4088/JCP.17r11506 29742331

[6] Leon SL, Cloutier P, Polihronis C, . Child and adolescent mental health repeat visits to the emergency department: a systematic review. Hosp Pediatr. 2017;7(3):177-186. doi:10.1542/hpeds.2016-0120 28196831

[7] Musey PI Jr, Schultebraucks K, Chang BP. Stressing out about the heart: a narrative review of the role of psychological stress in acute cardiovascular events. Acad Emerg Med. 2020;27(1):71-79. doi:10.1111/acem.13882 31675448 PMC7640378

[8] Calvano C, Warschburger P. Chronic abdominal pain in children and adolescents: parental threat perception plays a major role in seeking medical consultations. Pain Res Manag. 2016;2016:3183562. doi:10.1155/2016/3183562 28003776 PMC5143725

[9] Tanabe P, Persell SD, Adams JG, McCormick JC, Martinovich Z, Baker DW. Increased blood pressure in the emergency department: pain, anxiety, or undiagnosed hypertension? Ann Emerg Med. 2008;51(3):221-229. doi:10.1016/j.annemergmed.2007.10.017 18207606

[10] Musey PI Jr, Patel R, Fry C, Jimenez G, Koene R, Kline JA. Anxiety associated with increased risk for emergency department recidivism in patients with low-risk chest pain. Am J Cardiol. 2018;122(7):1133-1141. doi:10.1016/j.amjcard.2018.06.044 30086878

[11] Poulin PA, Nelli J, Tremblay S, . Chronic pain in the emergency department: a pilot mixed-methods cross-sectional study examining patient characteristics and reasons for presentations. Pain Res Manag. 2016;2016:3092391. doi:10.1155/2016/3092391 27829785 PMC5088325

[12] Krauss BS, Calligaris L, Green SM, Barbi E. Current concepts in management of pain in children in the emergency department. Lancet. 2016;387(10013):83-92. doi:10.1016/S0140-6736(14)61686-X 26095580

[13] Wilson JM; American Academy of Pediatrics Child Life Council and Committee on Hospital Care. Child life services. Pediatrics. 2006;118(4):1757-1763. doi:10.1542/peds.2006-1941 17015572

[14] Braun C, Stangler T, Narveson J, Pettingell S. Animal-assisted therapy as a pain relief intervention for children. Complement Ther Clin Pract. 2009;15(2):105-109. doi:10.1016/j.ctcp.2009.02.008 19341990

[15] Marcus DA, Bernstein CD, Constantin JM, Kunkel FA, Breuer P, Hanlon RB. Impact of animal-assisted therapy for outpatients with fibromyalgia. Pain Med. 2013;14(1):43-51. doi:10.1111/j.1526-4637.2012.01522.x 23170993 PMC3666031

[16] Barker SB, Dawson KS. The effects of animal-assisted therapy on anxiety ratings of hospitalized psychiatric patients. Psychiatr Serv. 1998;49(6):797-801. doi:10.1176/ps.49.6.797 9634160

[17] Muñoz Lasa S, Máximo Bocanegra N, Valero Alcaide R, Atín Arratibel MA, Varela Donoso E, Ferriero G. Animal assisted interventions in neurorehabilitation: a review of the most recent literature. Neurologia. 2015;30(1):1-7. doi:10.1016/j.nrl.2013.01.012 23642347

[18] Lundqvist M, Carlsson P, Sjödahl R, Theodorsson E, Levin LÅ. Patient benefit of dog-assisted interventions in health care: a systematic review. BMC Complement Altern Med. 2017;17(1):358. doi:10.1186/s12906-017-1844-7 28693538 PMC5504801

[19] Havener L, Gentes L, Thaler B, . The effects of a companion animal on distress in children undergoing dental procedures. Issues Compr Pediatr Nurs. 2001;24(2):137-152. doi:10.1080/01460860118472 11817428

[20] McCullough A, Ruehrdanz A, Jenkins MA, . Measuring the effects of an animal-assisted intervention for pediatric oncology patients and their parents: a multisite randomized controlled trial. J Pediatr Oncol Nurs. 2018;35(3):159-177. doi:10.1177/1043454217748586 29268667

[21] Gaudet LA, Elliott SA, Ali S, . Pet therapy in the emergency department and ambulatory care: a systematic review and meta-analysis. Acad Emerg Med. 2022;29(8):1008-1023. doi:10.1111/acem.14421 34817908

[22] Pet Partners. Volunteer requirements. Accessed September 15, 2024. https://petpartners.org/volunteer/requirements

[23] McKinley S, Madronio C. Validity of the Faces Anxiety Scale for the assessment of state anxiety in intensive care patients not receiving mechanical ventilation. J Psychosom Res. 2008;64(5):503-507. doi:10.1016/j.jpsychores.2008.02.002 18440403

[24] McKinley S, Stein-Parbury J, Chehelnabi A, Lovas J. Assessment of anxiety in intensive care patients by using the FACES Anxiety Scale. Am J Crit Care. 2004;13(2):146-152. doi:10.4037/ajcc2004.13.2.146 15043242

[25] Perpiñá-Galvañ J, Richart-Martínez M. Scales for evaluating self-perceived anxiety levels in patients admitted to intensive care units: a review. Am J Crit Care. 2009;18(6):571-580. doi:10.4037/ajcc2009682 19880959

[26] Jessop DS, Turner-Cobb JM. Measurement and meaning of salivary cortisol: a focus on health and disease in children: review. Stress. 2008;11(1):1-14. doi:10.1080/10253890701365527 17853059

[27] Barker SB, Knisely JS, McCain NL, Best AM. Measuring stress and immune response in healthcare professionals following interaction with a therapy dog: a pilot study. Psychol Rep. 2005;96(3, pt 1):713-729. doi:10.2466/pr0.96.3.713-729 16050629

[28] Kline JA, Fisher MA, Pettit KL, Linville CT, Beck AM. Controlled clinical trial of canine therapy versus usual care to reduce patient anxiety in the emergency department. PLoS One. 2019;14(1):e0209232. doi:10.1371/journal.pone.0209232 30625184 PMC6326463

[29] Gupta SK. Intention-to-treat concept: a review. Perspect Clin Res. 2011;2(3):109-112. doi:10.4103/2229-3485.83221 21897887 PMC3159210

[30] Sobo EJ, Eng B, Kassity-Krich N. Canine visitation (pet) therapy: pilot data on decreases in child pain perception. J Holist Nurs. 2006;24(1):51-57. doi:10.1177/0898010105280112 16449747

[31] Kertes DA, Liu J, Hall NJ, Hadad NA, Wynne CDL, Bhatt SS. Effect of pet dogs on children’s perceived stress and cortisol stress response. Soc Dev. 2017;26(2):382-401. doi:10.1111/sode.12203 28439150 PMC5400290

[32] Carey B, Dell CA, Stempien J, . Outcomes of a controlled trial with visiting therapy dog teams on pain in adults in an emergency department. PLoS One. 2022;17(3):e0262599. doi:10.1371/journal.pone.0262599 35263346 PMC9064456

[33] Nahm N, Lubin J, Lubin J, . Therapy dogs in the emergency department. West J Emerg Med. 2012;13(4):363-365. doi:10.5811/westjem.2011.5.6574 22942937 PMC3421977

[34] Benarous X, Milhiet V, Oppetit A, . Changes in the use of emergency care for the youth with mental health problems over decades: a repeated cross sectional study. Front Psychiatry. 2019;10:26. doi:10.3389/fpsyt.2019.00026 30787886 PMC6372506

[35] Wolyniez I, Rimon A, Scolnik D, . The effect of a medical clown on pain during intravenous access in the pediatric emergency department: a randomized prospective pilot study. Clin Pediatr (Phila). 2013;52(12):1168-1172. doi:10.1177/0009922813502257 24028842

[36] Sinha M, Christopher NC, Fenn R, Reeves L. Evaluation of nonpharmacologic methods of pain and anxiety management for laceration repair in the pediatric emergency department. Pediatrics. 2006;117(4):1162-1168. doi:10.1542/peds.2005-1100 16585311

[37] Trottier ED, Ali S, Le May S, Gravel J. Treating and reducing anxiety and pain in the paediatric emergency department: the TRAPPED survey. Paediatr Child Health. 2015;20(5):239-244. doi:10.1093/pch/20.5.239 26175559 PMC4472050

[38] Hartling L, Newton AS, Liang Y, . Music to reduce pain and distress in the pediatric emergency department: a randomized clinical trial. JAMA Pediatr. 2013;167(9):826-835. doi:10.1001/jamapediatrics.2013.200 23857075

[39] Antonioli C, Reveley MA. Randomised controlled trial of animal facilitated therapy with dolphins in the treatment of depression. BMJ. 2005;331(7527):1231. doi:10.1136/bmj.331.7527.1231 16308382 PMC1289317

[40] Ulrich RS. View through a window may influence recovery from surgery. Science. 1984;224(4647):420-421. doi:10.1126/science.6143402 6143402

