# Supplementary material for: Therapy Dogs for Anxiety in Children in the Emergency Department: A Randomized Clinical Trial
Source: JAMA Netw Open. 2025 Mar 14;8(3):e250636. doi: 10.1001/jamanetworkopen.2025.0636 (PMC11909607; doi:10.1001/jamanetworkopen.2025.0636)
Supplement: Supplement 1. — Trial Protocol [file jamanetwopen-e250636-s001.pdf]

Canine-Assisted ANxiety reduction IN Emergency care (CANINE): CANINE III

Heather Kelker MD

Principal investigator

SIGNIFICANCE

Prior literature demonstrates that human stress can be reduced with exposure to animals. (1-3) Other studies have found reduction in stress using therapy dogs in multiple healthcare settings.(3, 4) The benefit of therapy dogs in Emergency Medicine is novel and was explored in our first study, CANINE I. We found that adult patients with moderate to high anxiety in an Emergency Department setting had a 40% reduction in overall perceived stress after interacting with a therapy dog for 15 minutes. We now seek to explore the effect of canine intervention on stress levels in Pediatric Emergency Department patients. Prior studies have discussed children reporting a reduction in pain with the presence of an animal. Additionally, the positive effects of animal interaction have been observed in children and adolescents hospitalized for acute mental disorders, reducing emotional and behavioral symptoms and increasing global competence and psychological functioning. (10) One unpublished study has suggested that 3% of children are physically restrained during their stay in the Emergency Department and many more receive chemical sedation (Agraharkar, S et al). Interaction with therapy dogs may reduce anxiety and psychological distress, leading to lower need for pain medication and chemical and physical restraints. (2, 5)

RATIONALE

This study challenges current dogma by introducing a widely available, low-cost method of dog therapy to reduce patient stress. The organization "Paws of Love" estimates that it has 180,000 volunteers who have qualified therapy dogs and who are generally willing to volunteer their time in emergency care. The benefits may include improved perception of wellness, less opioid use, and decreased use of physical and chemical restraints. As a further extrapolation, this secondary effect may extend to improved patient-doctor communication and patient experience. This will in turn improve patient safety in the Emergency Department, decreasing the number of adverse events, and decreasing the risk of medical malpractice by improving the patient-provider relationship. (6, 7)

HYPOTHESES

The main study hypothesis is that pediatric patients with moderate to high anxiety will have a measurable reduction in stress and anxiety after interacting with a therapy dog, compared with usual care.

SPECIFIC AIMS

Compare the effect of a single exposure to a therapy dog and handler within the pediatric emergency department patient with anxiety by measuring the change in patient

perception of anxiety before and after dog exposure using the FACES scale and salivary cortisol level.

Exploratory aims

Test if a single exposure to a therapy dog and handler reduces parent perception of his or her child's anxiety, depression, and pain using the FACES scale. Test if a single exposure to a therapy dog and handler reduces parental anxiety by measuring the change in salivary cortisol level in one parent/guardian or other adult who will care for the child in the subsequent week.

Measure subsequent use of medications to alleviate anxiety and pain.

Record frequency of use of physical restraint.

INCLUSION AND EXCLUSION CRITERIA

All study procedures will be performed in the emergency department at IUH Riley Children's Hospital. Selection of participants for research will occur after patients and families have been qualified by a member of the Riley ED care team as eligible, according to standard procedures, for therapy dog visits at Riley hospital.

Inclusion criteria

- 5-17 years of age
- Emergency physician or nurse with primary care responsibilities for the patient agrees that the patient has "moderate or greater anxiety" (6 or greater on FACES scale)

Exclusion Criteria

- Violent behavior
- Any reported prior fear or adverse reaction to dogs in the dog intervention group
- Dog allergy, chief complaint asthma exacerbation with dog as known trigger

ENROLLMENT/RANDOMIZATION

Participants will be recruited by signed informed consent and authorization form solicitation by qualified study personnel. Patients will be enrolled during times when a hospital certified therapy dog and handler are and are not present. Study personnel will explain the study to the patient in the patient's room. After signed consent and authorization form, the patient and their parent(s)/guardian will complete the baseline (T0) FACES scales of anxiety/pain and salivary cortisol sample. [1,2] Patients who score a 0 on anxiety will be considered screen failures. Study design:

Block allocation: We will compare patient anxiety change based upon a predefined schedule of 2-hour blocks either with or without a therapy dog.

An example week of study:
Monday 4-6 pm No dog; 6-8 pm +Dog
Tuesday 6-8 pm No dog
Wednesday 4-6 pm + Dog; 6-8 pm No Dog
Thursday 6-8 pm +Dog
Friday 6-8 pm No Dog

	**Dog**	**Usual Care**
T0	FACES	FACES
	Salivary Cortisol	Salivary Cortisol
	Vital Signs*	Vital Signs*
	Demographics*	Demographics*
T1		
	Dog + Handler	
(~30-45 min after T1) PRIMARY AIM	FACES	FACES
	Salivary cortisol	Salivary cortisol
	Vital Signs*	Vital Signs*
T2 (as close to disposition as possible)	FACES	FACES
	Salivary cortisol	Salivary cortisol
	Vital Signs*	Vital Signs*

* Obtained from EMR when possible

To understand more about the quality of the interaction between the dog, handler and the patient, we will ask handlers and study personnel who may observe the interaction to keep field notes after sessions. They will be instructed to comment on verbal statements made by the patient or family that they believed were important. We will ask specifically for their impression on whether the patient touched the dog, facial affect changes and other non-verbal communication made by the patient. We will also ask handlers to assess the degree of dog-patient interaction using a series of questions and a Likert scale.

STATISTICAL CONSIDERATIONS

The primary outcome measure for the specific aim is the change in reported anxiety on the FACES scale from T0 to 30-45 min after T1, with the assumption that data will be normally distributed, and the means will be the same at baseline. Therefore, an unpaired t-test would be applicable. Extrapolating from our work in adults we set the clinically significant reduction in anxiety as requiring a

greater than 2 point (20%) decrease in anxiety 30-45 min after T1 compared with usual care, expecting a standard deviation of 3. (8, 9) With $\alpha=0.05$ and $\beta=0.20$, this required 37 pairs. Accordingly, the sample was set at 40 per group with complete data.

Per protocol analysis: Salivary cortisol and data from exploratory aims are tested by comparing mean or median values of the change in scales from T0 to 30-45 min after T1 and T2 (T1-T0 or ΔT) between the two interventions. Depending upon normality, we will either use an unpaired t-test or Mann Whitney U test to compare mean or medians from ΔT and to compare the mean or medians of T1. To examine for within group changes from T0 to 30-45 min afterT1, we will use either a paired t-test or a Wilcoxon rank sum test. If data are normally distributed or can be appropriately transformed to normal, we will also perform a 2-way repeated measures ANOVA to compare between groups for the change in perceived FACES scales. We will measure strength of correlation between patient and physician perceptions on the FACES scales with a Pearson's correlation coefficient or Spearman's rank coefficient as appropriate. We will compare dichotomous data such as the proportion of patients with a ≥2.5 difference in FACES, medication use and frequency of restraints with a Fisher's exact test or Chi Square statistic as appropriate. In instances where the patient is discharged from the emergency department prior to obtaining T2 measurement, the difference from T0 to 30-45 min after T1 will be used

We will report the results of field notes in accordance with Field notes from dog handlers and will be analyzed using commercial software (Nvivo Version 12.0.0.71, QSR International) to search for themes in patient verbal communication, changes in patient affect and changes in patient behavior from the start of the session to the end of the session. (19)

RECORD RETENTION AND PRIVACY

We will keep de-identified data for three years. Data will not be able to be re-identified to subjects.

References

1. Braun C, Stangler T, Narveson J, Pettingell S. Animal-assisted therapy as a pain relief intervention for children. Complementary therapies in clinical practice. 2009;15(2):105-9.

2. Marcus DA, Bernstein CD, Constantin JM, Kunkel FA, Breuer P, Hanlon RB. Impact of animal-assisted therapy for outpatients with fibromyalgia. Pain medicine (Malden, Mass). 2013;14(1):43-51.

3. Barker SB, Dawson KS. The effects of animal-assisted therapy on anxiety ratings of hospitalized psychiatric patients. Psychiatric services (Washington, DC).

1998;49(6):797-801.

4. Munoz Lasa S, Maximo Bocanegra N, Valero Alcaide R, Atin Arratibel MA, Varela Donoso E, Ferriero G. Animal assisted interventions in neurorehabilitation: a review of the most recent literature. Neurologia (Barcelona, Spain). 2015;30(1):1-7.

5. Havey J, Vlasses F, Vlasses P, Ludwig P, Hackbarth D. The Effect of Animal-Assisted Therapy on Pain Medication Use After Joint Replacement. Anthrozoos. 2014;27:361-9.

6. Smith DD, Kellar J, Walters EL, Reibling ET, Phan T, Green SM. Does emergency physician empathy reduce thoughts of litigation? A randomised trial. Emergency medicine journal : EMJ. 2016;33(8):548-52.

7. Kelm Z, Womer J, Walter JK, Feudtner C. Interventions to cultivate physician empathy: a systematic review. BMCMedEduc. 2014;14:219.

8. McConville J, McAleer R, Hahne A. Mindfulness Training for Health Profession Students-The Effect of Mindfulness Training on Psychological Well-Being, Learning and Clinical Performance of Health Professional Students: A Systematic Review of Randomized and Non-randomized Controlled Trials. Explore (New York, NY). 2017;13(1):26-45.

9. Mantzios M, Giannou K. When Did Coloring Books Become Mindful? Exploring the Effectiveness of a Novel Method of Mindfulness-Guided Instructions for Coloring Books to Increase Mindfulness and Decrease Anxiety. Frontiers in psychology. 2018;9:56.

10. Breisford V, Meints, K, Gee N, Pfeffer K. Animal-Assisted Interventions in the Classroom-A Systematic Review. Int J Envion Res Public Health. 2017;14(7): 669.

Additional references

1. McKinley S, Madronio C. Validity of the Faces Anxiety Scale for the assessment of state anxiety in intensive care patients not receiving mechanical ventilation. *Journal of psychosomatic research.* 2008;64(5):503-507.

2. McKinley S, Stein-Parbury J, Chehelnabi A, Lovas J. Assessment of anxiety in intensive care patients by using the Faces Anxiety Scale. *American journal of critical care : an official publication, American Association of Critical-Care Nurses.* 2004;13(2):146-152.
