# Supplementary material for: Therapy Dogs for Anxiety in Children in the Emergency Department: A Randomized Clinical Trial
Source: JAMA Netw Open. 2025 Mar 14;8(3):e250636. doi: 10.1001/jamanetworkopen.2025.0636 (PMC11909607; doi:10.1001/jamanetworkopen.2025.0636)
Supplement: Supplement 2. — eTable. Salivary Cortisol Measurements (μg/dL) [file jamanetwopen-e250636-s002.pdf]

Supplemental Online Content

Kelker H, Siddiqui HK, Beck AM, Kline JA. Therapy dogs for anxiety in children in the emergency department: a randomized clinical trial. *JAMA Netw Open*. Published online March 14, 2025. doi:10.1001/jamanetworkopen.2025.0636

eTable. Salivary Cortisol Measurements (µg/dL)

This supplemental material has been provided by the authors to give readers additional information about their work.

© 2025 Kelker H et al. *JAMA Network Open*.

eTable. Salivary Cortisol Measurements (μg/dL)					
Time	Dog, mean (SD)	Dog, mean change (T0-T1)	No Dog, mean and (SD)	No Dog mean change (T0-T1)	P value for change**
Child					
T0	0.19 (0.16)		0.19 (0.23)		
T1	0.15 (0.09)	0.044	0.16 (0.15)	0.027	0.74
T2	0.20 (0.21)		0.16 (0.12)		
Parent					
T0	0.57 (0.74)		0.74 (1.04)		
T1	0.42 (0.51)	0.177	0.40 (0.40)	0.214	0.71
T2	0.49 (0.59)		0.68 (0.96)		
*"Dog" denotes the handler and dog ; **Unpaired t-test applied to the T0-T1 change in Dog vs. No Dog					

© 2025 Kelker H et al. *JAMA Network Open*.
