# Supplementary material for: Therapy Dogs for Anxiety in Children in the Emergency Department: A Randomized Clinical Trial
Source: JAMA Netw Open. 2025 Mar 14;8(3):e250636. doi: 10.1001/jamanetworkopen.2025.0636 (PMC11909607; doi:10.1001/jamanetworkopen.2025.0636)
Supplement: Supplement 3. — Data Sharing Statement [file jamanetwopen-e250636-s003.pdf]

Data Sharing Statement

Kelker. Therapy Dogs for Anxiety in Children in the Emergency Department. *JAMA Netw Open*. Published March 14, 2025. doi:10.1001/jamanetworkopen.2025.0636

Data
Additional Information: NCT03784573
Data available: Yes
Data types: Deidentified participant data
How to access data: jkline@wayne.edu
When available: beginning date: 12-01-2025

Supporting Documents
Document types: None

Additional Information
Who can access the data: Researchers
Types of analyses: Any
Mechanisms of data availability: discussion with authors and signed data sharing agreement
